# A Qualitative Assessment of a Training and Communication Intervention on Antibiotic Prescription Practices Among Health Workers and Outpatients at Public Health Facilities in Uganda

**DOI:** 10.1093/cid/ciad329

**Published:** 2023-07-25

**Authors:** David Kaawa-Mafigiri, Deborah Ekusai-Sebatta, Elizeus Rutebemberwa, Asadu Sserwanga, Freddy Eric Kitutu, James Kapisi, Heidi Hopkins, Olawale Salami, Juvenal Nkeramahame, Piero Olliaro, Philip Horgan

**Affiliations:** Department of Social Work and Social Administration, School of Social Sciences, Makerere University, Kampala, Uganda; Infectious Diseases Research Collaboration, Kampala, Uganda; Department of Health Policy, Planning and Management, Makerere University School of Public Health, Kampala, Uganda; Infectious Diseases Research Collaboration, Kampala, Uganda; Department of Pharmacy, Makerere University School of Health Sciences, Kampala, Uganda; Infectious Diseases Research Collaboration, Kampala, Uganda; London School of Hygiene & Tropical Medicine, London, United Kingdom; FIND, Geneva, Switzerland; FIND, Geneva, Switzerland; FIND, Geneva, Switzerland; International Severe Acute Respiratory and Emerging Infection Consortium, Pandemic Sciences Institute, Nuffield Department of Medicine, University of Oxford, Oxford, United Kingdom; FIND, Geneva, Switzerland; Nuffield Department of Medicine, Big Data Institute, University of Oxford, Oxford, United Kingdom; Evidence and Impact Oxford, Oxford, United Kingdom

**Keywords:** acute febrile illness, antimicrobial resistance, prescription adherence communication, prescription adherence behavior

## Abstract

**Background:**

Antibiotic prescribing practices are 1 of the contributing causes of antimicrobial resistance (AMR). The study explored the key drivers and barriers to adherence to prescribing instructions among healthcare workers and outpatient attendees with the aim of developing a training and communication intervention to improve adherence to prescription.

**Methods:**

Prior to randomized trials at 3 health centers in Uganda (Aduku, Kihihi, and Nagongera), a pre-intervention qualitative assessment was conducted to explore behavioral drivers for adherence to prescriptions and the communication of adherence messages. Based on the findings, a training and communication package was developed for healthcare workers and patients at Day 0 of the trial. During the trial's Day 7 patient follow-up, in-depth interviews were conducted to further investigate adherence behaviors.

**Results:**

Five main themes were identified that acted as drivers or barriers to prescription adherence. Key drivers included: drug availability at health facility, health worker knowledge, and communication to patients. Barriers included: care-seeker use of treatment resorts and an inability by care-seeker to buy drugs.

**Conclusions:**

The T&C appeared to influence both health workers’ and patients’ behavior and improve adherence to prescription.

The adapted T&C should be considered a toolkit to improve antibiotic use across health facilities accompanied with appropriate guidelines to mitigate AMR.

## Teaser

Qualitative assessment of healthcare providers' prescription of and patients' adherence to antibiotics informed training and communication (T&C) for the Antimicrobial Resistance (AMR) Diagnostic Use Accelerator clinical trial in Uganda, indirectly reducing AMR through adherence messages to intervention arm participants.

The “just-in-case” antibiotic prescribing practice is thought to be a main cause of antimicrobial resistance (AMR) and inadequate management of acute febrile illnesses, both resulting in increased morbidity and mortality [1]. As such, AMR is recognized as a global public health problem, with antibiotic use rising considerably in recent years, particularly in low- and middle-income countries (LMICs) [2]. At the same time, many who require antibiotics are not prescribed them [3]. An adaptation in practice is therefore needed to improve the case management of acute febrile illnesses in children and adolescents presenting to outpatient clinics or peripheral health centers in LMICs.

To achieve this, FIND's, AMR Diagnostic Use Accelerator program (protocol https://pubmed.ncbi.nlm.nih.gov/33239106/ [4]) was initiated in 3 African countries (Burkina Faso, Ghana, and Uganda) to investigate the impact of an intervention package consisting of diagnostic tools, clinical algorithms, clinical processes, and a training and communication (T&C) package on (1) antibiotic prescriptions in children and adults presenting with fever, and (2) clinical outcomes, compared with routine clinical practice. As part of this program, the Advancing access to Diagnostic Innovation essential for UHC and AMR Prevention (ADIP) randomized controlled clinical trial was conducted across three health centers in Uganda. As part of the trial, and prior to the clinical intervention, a qualitative research study was implemented, resulting in a T&C package used in the clinical intervention. The main aims of this qualitative and behavioral framework study were to investigate (1) the social, economic, and cultural factors that supported or hindered patients” adherence to prescriptions and (2) the communication of adherence messages (including behavioral determinants) from healthcare workers to patients/caregivers. Here, we present the findings from this qualitative study; the quantitative findings from the randomized clinical trial are reported separately (please see Kapisi et al in this supplement).

## METHODS

The study comprised a baseline (pre-intervention) and post-intervention phase. It also allowed for the measurement of the effects of the intervention on the behaviors of patients from the surrounding communities.

### Study Sites

The study was conducted in the following 3 sites: Aduku health center IV, located in Aduku Sub-County in Kwania District (formerly Apac District) in mid northern Uganda; Kihihi Health center IV, located in Kihihi Sub County in Kanungu District in southwestern Uganda; and Nagongera, located in Tororo district in the eastern part of the country.

### Baseline Methodology

Baseline assessment was conducted, using focus group discussions (FGDs) and in-depth interviews (IDIs), between 23 February and 12 March 2020 across the 3 sites in Uganda. In this phase, the study aimed to (1) understand the contextual factors that affect patient/caregiver adherence to the prescription and (2) understand the factors that affect communication of adherence messages by the healthcare workers. The information that was collected in this phase was used to design 1 component of the intervention: the “Training and Communication package (T&C)” (please see supplementary material).

The study was guided by the interpretivist paradigm, which seeks to understand the subjective world of human experience. This approach makes an effort to “get into the head of the subjects being studied” and to understand and interpret what the subject is thinking or the meaning they make of the context [5].

### Sampling and Participants

Sampling for the FGDs was conducted by the health facility focal person, a village health team (VHT) member who liaises with the community as part of their routine public health tasks. The focal person selected adult male and female community members through purposive sampling, within the health facility's catchment area. The health focal person selected the participants from the respective households and considerations were made between those who stayed in townships and suburbs. The sample of the community members was stratified to cater for gender and age considerations of different patients.

Health workers whose role involved prescribing drugs and taking samples in the laboratory, were purposively sampled for IDIs.

Written consent was sought from all research participants prior to participating in the study. All those who signed consent were then registered on the enrollment log.

### Data Collection Methods and Instruments

To understand the contextual factors, barriers and facilitators to adherence to prescription, both FGDs and IDIs were conducted, by qualified social science research assistants recruited independently from the health facility staff. The interviewers were guided by a topical guide. The FGDs were conducted with patients and caretakers in the local languages of each district (Luo for Kwania District, Runyakitara for Kanungu District, and Dhopadhola for Tororo District). The IDIs were conducted in English with health workers. In total, 125 participants were involved in 12 FGDs (Table 1), and 18 IDIs were conducted (Table 2) across the 3 districts.

**Table 1.** Summary of FGDs Conducted by District and Characteristic of Participants (N = 12)

Participant Characteristic by FGD	Number of Participants by District/FGD			
	Nagongera	Aduku	Kihihi	Total Participants
FGD of patients (Male)	12	9	12	33
FGD of patients (Female)	10	10	11	31
FGD of caretakers (Female)	0	10	0	10
FGD of caretakers (mixed male/female)	8/2	0	5/5	20
FGD of household heads (male)	0	0	9	9
FGD of household heads (mixed male/female)	6/4	10/2	0	22
Total	**42**	**41**	**42**	**125**

Abbreviation: FGD, focus group discussions.

**Table 2.** Summary of IDIs Conducted by District and Participants’ Gender (N = 18)

Gender	Number of IDIs Conducted by District and Gender			
	Nagongera	Aduku	Kihihi	Total
Male	3	4	3	10
Female	3	2	3	8
Total	**6**	**6**	**6**	**18**

Abbreviation: IDI, in-depth interview.

Three social scientist research assistants competent in the predominant local languages at each study health center conducted the interviews. They were trained on the protocol and interviewing techniques by the qualitative study coordinator and the principal investigator.

Development of topic guides: A team-based approach was used in the development and refinement of the topic guides. The guide was modified for local context and language and shared with the social science field team for feedback.

### Data Management

All FGDs and IDIs were audio recorded. Audio recordings from the FGDs were transcribed and translated into English, whereas the IDIs were directly transcribed verbatim into English. All audio recordings were stored on the computer and an external hard drive as backup. All audio recordings were deleted from the audio recording devices after being transferred on to the computer.

### Analysis

A team-based approach to analysis was employed [6]. Content analysis was used to generate a coding frame and categories. A codebook was generated using content analysis and pilot-tested on 5 transcripts from the IDIs and 5 from the FGDs [7]. Three coders (D. E. S., D. K. M., E. R.) independently coded the responses. An independent checker (P. H.) resolved discrepancies between the 3 coders and provided insight on the way forward. Independent senior social scientists (S. N., C. N.) not directly involved in the study reviewed the code book and provided feedback which improved the quality of the findings. Analysis was done both manually and by the use of NVivo version 12 software to develop overarching themes of the analysis. The software was used in the organization and analysis of the data.

Confidentiality: Participants were assigned unique study identifier numbers and informed that their personal study-related data would be used by the study team and sponsors.

### T&C Intervention Phase

The T&C was 1 component of the intervention package of the AMR Diagnostic Use Accelerator clinical trial in which patients were randomized to either the intervention or control arm. From the findings, a T&C package was developed and pretested to ensure clarity. In addition, the T&C package was then reviewed by a communications expert to refine how best the messages could be communicated to the patients. For instance, the use of pictures, as well as simple, concise statements were adopted to communicate the messages clearly. The communications expert helped to identify some relevant pictures that were relatable to the study context.

Health workers in the intervention arm (hired by the study team) used the T&C package with only the intervention arm patients, whereas health workers in the control arm (staff employed by the participating health facilities) carried out the standard of care. Further details of trial design are described in this supplement (Kapisi et al).

Health workers were trained on the T&C and guided role plays were done among them to boost confidence. The T&C was pretested for clarity with sample patients.

At Day 0 patients who were recruited into the study met with the social scientist who assessed their current health care seeking practices. They were then sent to the study clinician who took them through the T&C. Simple messages were developed for use in the T&C.

The trained healthcare workers were asked not to discuss the T&C information with the non-trained healthcare workers, to reduce contamination.

### Day 7 Methodology

The social scientists also met with both intervention and control arm patients on Day 7 to assess their adherence to the prescription received on Day 0.

Analysis of Day 7 transcripts from both the control and intervention arms was carried out using a similar methodology as described above for the baseline study, and used to identify key themes and concepts generated from the intervention. In total, 75 participants were purposively sampled and recruited to participate in either the control or the intervention arms (Table 3).

**Table 3.** Summary of Day 7 IDIs Conducted by District and Trial Arm Category

Clinical Trial Arm	Number of IDIs Conducted in Day 7 by District and Trial Arm			
	Nagongera	Aduku	Kihihi	Total
Control arm participants	10	12	19	41
Intervention arm participants	11	12	11	34
Total	**21**	**24**	**30**	**75**

Abbreviation: IDI, in-depth interview.

## RESEARCH FINDINGS

### Baseline Results: Focus Group Discussions (FGDs)

The behavioral drivers identified in the baseline analysis that act as barriers and enablers to prescription adherence are summarized in Tables 4 and 5 and described in detail below.

**Table 4.** Key Thematic Areas From the Findings of Focus Group Discussions With Community Members, Stratified by Health Center Factors and Patient Factors

	Barriers	Drivers
1. Health center factors		
Health worker knowledge, attitude, and skills	No communication to patients, rude health workers, inexperienced, don't keep time	Communication about adherence to prescription, positive attitude, approachable
Drug availability	Treatment resorts like use of herbal medicine, sharing medicine	Drug availability at health center, influences ability to take drugs on time and completion
Tests performed	Shortage of kits to perform the required tests	Availability of tests, health worker skills to perform the tests
2. Patient factors		
Patient factors	Self-medication, buying a small dose, sharing drugs	Completion of prescribed drug regimens
Support network	Sharing medicine, no treatment companion	Treatment support, support in going to the health center, influence of opinion leaders
Financial support	No capacity to buy drugs or travel to the health center	Access to the facility and drugs

**Table 5.** Overarching Themes—Barriers and Drivers

Themes	Barriers	Drivers
Drug availability at health facility	Treatment resorts, sharing medicine	Taking drugs on time and completion
Patient factors	Self-treatment, buying a small dose, sharing drugs	Completion of drugs
Health worker knowledge, attitude and skills	No communication to patients, rude health workers, inexperienced, don't keep time	Communication about adherence to prescription, positive attitude
Patient support network	Sharing medicine	Treatment support, support in going to the health center, influence of opinion leaders
Patient financial capacity	No capacity to buy drugs or travel to the health center	Access to the facility and drugs

### Patients/Caretakers’ Perspectives of the Effect of T&C

Overall, the T&C package appears to have a positive effect on patients/care takers’ adherence practices as discussed below:

### Improvement in Practices that Affect Adherence

#### Poor Treatment Practices

Prior to the T&C, many respondents noted having difficulty following the instructions recommended by the health workers. A lack of appreciation of the need to complete the prescribed dose of medicine was noted, with several FGD respondents who reported abandoning doses given to them once they felt better and only resuming medication if their symptoms reoccurred. Participants also experienced different circumstances that affected how they took prescribed medicines. These included starting a dose at a later time than was recommended by the health worker, forgetting or skipping doses altogether, while others mentioned doubling the dose the next time it was due to be taken.

“When I am given medicine and I forget, let's say I take in the morning and it is supposed to be taken at 1 and 2 Pm, no no no no, 2 Pm is too near. And maybe 4pm or in the evening and I forget and maybe travel then I take that of morning and leave others and start the next day, and this time I be serious, and I follow the prescription.” (FGD with women)

Other poor prescription-related practices noted were sharing of drugs such as among family members, inability to demonstrate knowledge or understanding of dose regimens, self-medicating during a subsequent episode of illness when symptoms were perceived to be similar to the previous episode for which treatment had been sought, and utilization of multiple treatment resorts including simultaneously taking drugs with herbs or other medication outside the one prescribed at a health facility.

Community member FGD participants reported that the T&C was useful in educating them about the above-mentioned practices that affect/hinder adherence to appropriate care.

#### Pluralistic Treatment Resort

One major area that patients/caretakers reported learning about was the importance of refraining from multiple, sometimes simultaneous treatment resorts like taking herbal medicine in place of, or together with the prescribed medicine. Following the T&C, patients/caretakers reported ceasing to purchase drugs that were not prescribed to them at the facility where they sought care.

“We are happy with the information that the basawo (clinicians) give us because initially we would always use budomola (herbal medicine) for treatment of anything and we would come to the health center after it had failed to work. But now we understand that is always good to first come to the hospital and avoid self-medication.” (Patient HC IV)

#### Incomplete and/or Wrong Dosage

Similarly, patients reported having learned the dangers of sharing medicine, getting incomplete doses and self-medication which was considered a waste of resources most of the time. Some patients/caretakers previously did not know how to describe the meaning of when to take their dosage and as such may have been taking the medication wrongly. Some were unable to repeat the dosage instructions accurately whereas others simply misinterpreted it.

The T&C demonstrated the importance of health worker communication with the patients in simplified language. Following the T&C, patients/caretakers reported learning how to describe their prescriptions. Some were able to repeat the type of medication they were given and the frequency with which they were to take their medication. For instance, the frequency was now articulated in terms of counting hours compared to the past when they described it in terms of the time of day (morning, afternoon, evening), the ability to mention the color of the drugs, and articulate several ways to take the medication. Some patients/caretakers reported that they were now able to understand the doctors’ instructions and directives not to buy additional drugs outside of the prescribed ones. Respondents also understood the importance of returning the pill containers and pill balances when returning to the health facility compared to previously when they would “store them for later use.”

“Yes, I was able to make sure I follow the prescription I am told to give as written for me and also able to learn that it is important for me to keep time when giving the medicines.” (Patient HC IV)

#### To Test Before Treatment

Some patients/caretakers opined about the T&C being a driver for good adherence as it helped them better appreciate the need to first seek consultation at a facility. They reported more awareness about diagnostic tests, including the importance and role of a number of investigations to determine the cause of illness prior to taking any medication:

“I was able to learn that it is of great importance to first come for testing before I swallow medicine, which was not the case with me before.” (Patient Health center IV)

“I was able to understand the importance of first bringing my child to hospital for testing before giving any medicine, not just buying here and there medicine and giving to my child, which has not been of help to my child.” (Care giver Health center IV)

#### Failure to Follow Prescriptions

When narrating their experience at the health facility, community members cited drug stockouts as a major challenge to adhering to prescriptions. The regular drug stockouts at the public health facilities required them to purchase the prescribed medicines from private providers. The challenge, however, is that the majority of the patients could not afford the medicines sold at high cost in private clinics. When community members were able to access the medicines, they did not always take the medicines as prescribed by the health workers. As such participants resorted to several patterns to cope with the stockouts and unaffordable alternatives including postponing the start of a dose, suspending treatment or stopping the treatment completely for a variety of reasons. The majority of the respondents reported at baseline that the requirements set by the health workers were difficult to follow and were “beyond their means” as is described by a female patient. A lack of appreciation to continue the dose was noted with several FGD respondents. They reported abandoning doses given to them once they felt better and only resuming taking the medicine if their symptoms recurred.

“The problem is many times when you feel better, you become lazy to complete the dose because sometimes you don't even have money to buy the full dose yet; the basawo (health workers) always tell us that we have to complete.” (FGD Women)

Notably, community members experienced different circumstances that affected how they took prescribed medicines. During baseline interviews, the majority of participating community members mentioned starting a dose at a later time than was recommended by the health worker, citing lack of money to purchase the drugs at the recommended time. Several community members mentioned that if a dose was forgotten, it was skipped altogether, whereas others suggested doubling the dose the next time it was due to be taken.

“Because of our busy schedules, as men most times you end up forgetting to take the medicine and when you remember sometimes it is too late and you just decide to just miss the pill.” (FGD male group)

“As a woman and the mother of a child, we fear too much so when you remember that you forgot to give the child medicine, most times we either give any time we remember or double the next dose.” (Caregiver HC IV)

Participants reported during Day 7 interviews that the above concerns were now better appreciated with less likelihood to continue those inappropriate prescription behaviors in part due to the knowledge gained from the T&C intervention.

#### Health Worker Perspectives

The health workers thought the T&C had played a great role in influencing both the health worker and patient behavior.

#### Communicating to Patients

The health workers were trained on use of the T&C which sought to equip them with skills on how to communicate effectively with the patients. The health workers understood the importance of having a right attitude in treatment of the patients. The T&C placed emphasis on the importance of communicating to the patients in a simplified way, in a language understandable to the patients and importantly explaining to them how to swallow their drugs.

“Like I said earlier we were trained, when initiating the study we were taken out for training. So, I will tell the patient how we treat according to the results and what we use for treating. For example, if the child has malaria, I will say your child will need 1 tablet in the morning or 1 tablet in the evening. I will then ask the patient if they have understood and ask them to repeat back what I said, and I correct the wrong information.” (Health worker female)

#### Prescription based on the Test Results

T&C provides the health workers knowledge on the importance of prescribing medication based on the available results. The health workers in the intervention arm are availed with a wide range of tests and patients are talked to during the T&C about the benefits of prescribing basing on the results in getting proper diagnosis.

The T&C has not only changed the patient attitude but has also influenced the health worker attitude regarding the importance of testing. One health worker opined:

“I am a parent myself. I have children and previously I would do the other way (treating without testing), but now it has shaped me I know that not every fever or every cough needs an antibiotic. It has started with me. Recently I was home, and I found my son having fever, was having a cough, I had to go to the hospital lined up and did the tests.” (Male health worker)

#### Behavior Change of the Patients

The T&C has brought about change in patient attitude and perception towards misuse of antibiotics. Some patients previously thought they required antibiotics when they didn't feel well. The health workers explained to the patients that they treat based on the results and the given results showed they did not require some antibiotics.

“There is a way it has modified people's reasoning, people's perception towards these antibiotics. I have living examples where patients have come back testifying that ‘Do you know what, doctor, do you remember the other day when I was insisting on taking some medicine and you said no. I tried your advice and I have to say that I lost it and you won.’ I didn't actually take the medicine, but I feel I am okay.” (Male health worker)

A majority of the health workers reported that communicating about adherence to prescribed drugs would result in patients recovering well upon completion of the prescription. Other health workers thought that communication about adherence would help to minimize potential for antimicrobial resistance, which would have come due to inappropriate use of antibiotics. In the quote below, a doctor describes the benefits of communicating well about adherence.

“But when we communicate well, we are likely to see reduced costs because the patient is going to take drugs for 3 days, and the patient will be well because he has taken them on time and does not need additional drugs. Then we are likely to reduce the incidences of resistance, and we are likely to reduce multi-drug cross-resistance.” (Doctor HC IV)

Several challenges in communicating adherence were noted. The large patient load seen at the health facility was mentioned by majority of health workers as a barrier to communicating effectively to patients. Health workers admitted that sometimes they were focused on clearing the long queues and paid less attention to the information they gave patients. The long patient queues were partly attributed to the low staffing levels at health facilities, which caused the few health workers to work under pressure.

“Then in outpatient the problem might be because of the numbers. Then the nurse will give the information very fast. Not giving very slowly so that she reaches to the point that the patient understands, yet this is an old man who does not know 3 times a day.” (Clinician HC IV)

Health workers also reported having little control over what the patients did, specifically if there were stockouts. Prescribing the right dose to be taken was not always a guarantee to the health worker that the prescription would be followed. They expressed that once the patient left the health facility and went to the private facilities, they could not ascertain whether the prescription was purchased as was given and therefore could not be sure if the actual prescription was taken.

“Ok, one of the challenges that we face is availability of drugs. Because if I am telling you to swallow a drug that is taken twice a day and the drug is not here, that means you are going to go to the pharmacy or outside to the clinic. So, whatever they tell you from there, now what? I have no control.” (Clinician HC IV)

### Intervention Day 7 Findings

Across all 3 study sites, analysis of the Day 7 interviews among both males and females identified the following key drivers that support prescription adherence: drug availability at the health facility, health worker knowledge, communication, and opportunities such as a good patient support network, the capability of the health workers with good knowledge, health worker cognition, and interpersonal skills. Additionally, both health workers’ and patients’ motivation and awareness from the T&C package appeared to be influenced by positive social and environmental factors to tackle poor adherence.

The T&C package created awareness about the need to adhere to the prescribed medication. Participants were made aware of the benefits of testing and the dangers of self-medication in creating antimicrobial resistance. The research participants were made aware that even when they did not feel well, they did not always require some form of antibiotics. The T&C was useful in educating the participants about the importance of refraining from treatment resorts like taking herbal medicine in place of the prescribed medicine, the dangers of sharing medicine, getting incomplete doses, and self-medication, which was considered a waste of resources most of the time.

“The T&C has been a new package from the time we started the intervention patients, I don't want to say that 100%, it has changed every one we have given it to. However, there is a way it has modified peoples reasoning, people's perception towards these antibiotics. I have living examples where patients have come back testifying that “do you know what doctor; do you remember the other day when I was insisting on you giving me some medicine? I tried your advice, and I didn't take the medicine, but I feel I am okay.” (Clinician HC IV)

The T&C package demonstrated the importance of health worker communication to the patients in simplified language. The T&C package enabled participants to repeat back the type of medication they were given and the frequency in which they were to take their medication. Respondents also understood the importance of returning the pill containers and the pill balances when returning to the health facility.

Some patients opined about the T&C being a driver for good adherence:

“I was able to learn that it is of great importance to first come for testing before I swallow medicine, which was not the case with me before.” (Patient Health center IV)

“I was able to understand the importance of first bringing my child to hospital for testing before giving any medicine not just buying here and there medicine and giving to my child, which has not been of help to my child.” (Caregiver Health center IV)

### Barriers to Adherence

A key barrier to adherence was the drug stock out at the health facilities attributed to the large patient loads. This affects planning, leaving health facilities with insufficient drugs, only enough to meet the needs of a small proportion of patients. Health workers have no choice but to prescribe the drugs for the patients in their books so that they can be bought from outside the health facility, mainly from nearby drug shops. Majority of the patients who seek care from the health center IVs are of low- and middle-income status, who occasionally walk to the facilities because they cannot afford the transport costs involved. Having to buy the drugs then becomes a challenge, and the patients resort to buying half dosage or sharing drugs with other family members, increasing the risk of antimicrobial resistance.

“Ok, one of the challenges that we face is actually availability of drugs. Because if I am telling you to swallow a drug that is taken twice a day and the drug is not here, that means you are going to go to the pharmacy or outside to the clinic. So, whatever they tell you from there, I have no control.” (Medical officer HC02)

Although the T&C appeared to positively influence patient adherence, structural factors such as the drug stock being out, which the health workers had no control over, negatively affected adherence.

Additionally, in some cases, some of the intervention patients who had been exposed to the T&C did not report good adherence at Day 7. For instance, in some circumstances, intervention patients were still influenced by dispensers at the drug shops when they went to buy their prescribed drugs.

“The cough linctus when I went to buy, the person who dispensed for me the medicine gave a tablet medicine instead of the syrup they had written for me. She said it was better.” (Female patient, IDI)

## DISCUSSION

The T&C package appears to have a positive effect on healthcare providers’ communication of the prescription and adherence messages, and patients'/caretakers’ practices of adherence to prescriptions. Notably, the analysis of quantitative data on prescription adherence for this study suggests a likely positive impact of the T&C on adherence to antibiotic prescriptions. Patients exposed to the T&C intervention had a large improvement in adherence to antibiotic prescriptions compared to those in the control arm (71.3%, 42.5%; please see Compaoré et al in this Supplement). Health workers reported that there was an improvement in adherence by patients/caretakers demonstrated by changes in practices like being able to describe how to take their prescriptions, being able to tell how to take the prescription either by the time of day, or mealtimes and by color schemes representing what drugs to take throughout the day. Additionally, health workers reported that patients/caretakers expressed their appreciation for the need to first undertake tests and other diagnostics before getting medication.

Relatedly, patients/caretakers reported having stopped several practices that contribute to non-adherence including sharing of medication; non completion of dosage; buying additional medication from drug stores that were not prescribed by health workers at the facilities they sought care from and returning unfinished dosage every time they visited the health facility for follow-up care.

Health workers also report that they were able to improve their messaging and education of the clients as the T&C was characterized as being “simple to deliver” in part due to the pictorials and simple language it adopts. This was welcomed by the health workers who perceived their patients/care takers to better understand their instructions and thus make their work easier in light of the heavy workload.

However, findings also revealed that there remain contextual factors beyond the agency of the patients/caretakers to improve adherence. These included the drug stockouts reported by participants from all 3 study facilities, which led them to purchase drugs from alternative sources like drug shops. While there they reported encountering situations where they were either given other prescriptions in addition to what they needed or other medications to supplement what they were prescribed at the health facilities. Moreover, patients/caretakers across all study sites also reported that sometimes they were unable to buy full doses due to the prohibitive costs, a practice that would likely lead to non-adherence as the medication may not be taken to completion. Patients/caretakers also appear to continue practicing medical pluralism where they seek alternative care including non-biomedical sources like herbs. The use of other complementary sources appeared to be triggered in part due to the social context in which care-seeking takes place.

The study was unable to examine the social context of each participant in-depth as it was not designed to do so. Moreover, the short interval between baseline and post-intervention (Day 7) assessment may not have provided extended exposure time to examine the effect of the intervention (T&C). Relatedly the study did not set out to specifically identify differences in barriers/enablers of adherence based on gender or geographic setting. Even then the results are still suggestive of the positive effect, at least in the short term, that the T&C has on adherence to prescriptions among both healthcare workers and their patients.

## CONCLUSION

This study demonstrated that the T&C package can be useful in bringing about behavior change and improving adherence to prescriptions among both healthcare workers and their patients. As such, this and adapted training and communication packages should be considered as part of the toolkit required to improve antibiotic use in the ongoing fight to mitigate the development and spread of AMR.

## Supplementary Data

Supplementary materials are available at *Clinical Infectious Diseases* online. Consisting of data provided by the authors to benefit the reader, the posted materials are not copyedited and are the sole responsibility of the authors, so questions or comments should be addressed to the corresponding author.

## Supplementary Material

ciad329_Supplementary_Data

## Notes

***Author Contributions.*** H. H., J. K., D. K. M., and P. H. were involved in conception, H. H., J. K., D. E. S., D. K. M., F. E. K., E. R., and P. H. were involved in the design, D. E. S., J. K., A. S., and D. K. M. were involved in data acquisition, D. E. S., E. R., D. K. M., and P. H. were involved in data analysis and interpretation. All authors reviewed the manuscript drafts and approved the final version.

***Acknowledgments.*** The authors acknowledge the support of the research participants, field social scientists, and the health facility in charge, who provided administrative approval, and the clinicians who took part in either the intervention or control arms of the study.

***Supplement sponsorship.*** This article appears as part of the supplement “Using Diagnostic Tools to Support Antimicrobial Stewardship and Improve Outcome in Resource-Limited Contexts,” sponsored by FIND.

***Ethics statement.*** The study obtained ethics approval from the Makerere University School of Biomedical Sciences Research and Ethics Committee (reference SBS 715) and national clearance from the Uganda National Council for Science and Technology (reference HS 2727), Oxford University (OxTREC reference 200128 52–19), and the London School of Hygiene & Tropical Medicine Research Ethics Committee (reference 26684). Trained study team members explained the nature of the study to each participant or parent/guardian in a language understandable to him/her and answer all questions about the study.

***Financial support*.** This part of the study was funded by the Swiss Agency for Development and Cooperation (SDC), the Federal Ministry of Economic Cooperation and Development (BMZ), and the UK Department for International Development (DFID), now Foreign, Commonwealth and Development Office, United Kingdom (FCDO).
