# Supplementary material for: A Qualitative Assessment of a Training and Communication Intervention on Antibiotic Prescription Practices Among Health Workers and Outpatients at Public Health Facilities in Uganda
Source: Clin Infect Dis. 2023 Jul 25;77(Suppl 2):S191–8. doi: 10.1093/cid/ciad329 (PMC10368412; doi:10.1093/cid/ciad329)
Supplement: ciad329_Supplementary_Data [file ciad329_supplementary_data.docx]

**Links between research findings and T&C package design**

efinitMessaging in view of health worker linked factors

| Research finding | What health workers need to know | Health workers’ role | Facilitators | Quote illustrating research finding |
| --- | --- | --- | --- | --- |
| Difficulties in communicating in adherence upon | - Communicating about adherence to prescribed drugs would result in patients recovering well upon completion of the prescription. - Communication about adherence helps to minimize potential for antimicrobial resistance which would have come due to inappropriate use of antibiotics. | - Ensure that the right prescription is given - Ensure that the patient recognises and understands the prescription given (how to take it, what to expect, side effects, drug interactions). - Talk to the patient about the importance of adherence (emphasize the benefits and clearly point to the dire consequences) | - Use personal examples to provide a vivid picture - Deliver the message in a language the patient or guardian understands (consider an interpreter where there is language barriers) - Ask to talk to a trusted person within the patient’s support network | - ***In response to barriers in communicating prescription to adherence*** - *R: When you are on duty and you are in a very sad mood, or you are very angered or rough, it can affect your communication. And the patient might not understand very well. Or every time you bark at the patient or you are barking at them, or you are hurrying and trying to rush them. They might not understand you very well (***Nursing officer, HC 01)** - R: Because, some of our patients or clients do not know English. Then you cannot talk to them in English you have to talk to them in rukyiga and explain to them properly so that they can understand (**IDI** **002, Nursing officer HC 03)** |
| Challenges of communicating adherence | - Large patient loads amidst low staffing levels affect the quality of information given to patients due to pressures to clear the queues | - At all times and amidst the challenges ensure that the patient gets the relevant information to facilitate adherence to prescription | - Sorting patients according to diagnosis and prescription and deliver a common message but emphasis put on the uniqueness in experiences | - *The numbers are big and it’s a challenge in administration. We had a case yesterday where someone was being rude to the patient and of course she was working more than the expected hours. She had worked more that the expected time and eventually started being rude to the patient. If I have been in a very demanding situation when you meet me I would have wrinkled my face”* ***IDI 001 Medical officer HC 03*** - “*The little time that we have with our patients, (I: hmmm.) We are always over whelmed with patients. (I: Hmmm.) Two, (I: the patients are more than the health workers) than the health workers”* **IDI 001 Nursing officer HC 01** |
| Little control over what patients do, specifically if there were stockouts | - Many factors will determine the next steps for the patient   - Messaging and reception of information about the stock out, knowledge access prices, access availability, affordability, preference, prices, and how this is delivered is crucial | - Provide an appropriate prescription - Ask the patient if there is a recognised facility with qualified physician near where they live to present the prescription - Try to understand the patient’s socio-economic status - Encourage them to provide feedback on the medication | - Work on the attitude of both the health worker and the patient especially at a time you are delivering the message on stockout (**See strategies for physician patient relationship)** - Remember both may be frustrated by stockout. Better to ensure that all are calm - Find out if there are recognised structures in the community that can follow-up to ascertain the right prescription was obtained and monitor anti-biotic use in children | *R: Ok, one of the challenges that we face is actually availability of drugs. Because if I am telling you to swallow a drug that is taken twice a day and the drug is not here, that means you are going to go to the pharmacy or outside to the clinic. So, whatever they tell you from* *there, I have no control (****IDI 003, medical officer HC02)***  *R: we always have drugs tailored to be at HC IV but these drugs only last for 2 weeks. We have bi-monthly drugs delivered; every 2 months they deliver drugs but these drugs last for two weeks. So, it means we have one and a half months without drugs. One you do not have drugs,*  *It takes a good heart for you to explain to somebody to understand you* ***(IDI 001, medical officer HC 003****)* |

**Messaging in view of care provider linked factors**

| Research finding | What patients need to be aware off | - Care provider/patient role | Facilitators | Quote illustrating research finding |
| --- | --- | --- | --- | --- |
| Social and economic pressure (competing needs) | - Feeling better before completing the prescribed regimen/dose does not mean healing | - Continue with medication to completion - Consider consulting with your physician on the next steps | - Inform your physician about the progress you are making | ***Challenges to following prescription***  *cR6: The doctors can prescribe for the child medicines and the child gets well. So, instead of completing the dosage, the caregiver may not continue ensuring that the child completes the dosage (****FGD4 Household HC,03).***  R1: *Sometimes you may visit the health centre with a severe health problem and you may require to take expensive medicine yet you don’t have the money to afford* **(FGD01 Care givers, HC01***).*  *R11: When I buy medicine and take half of it and feel better, I stop taking the medicine, I say let me pose the dose and go to attend to my gardens... when I dig and dig and feel some pain again, I blame the hoe yet I didn’t complete the dose, and I resort to local herbs. It is true I fear medicine. For me when I take local herbs I feel very fine (****FGD 01 patients HC003)*** |
| Responsibility to help others | - A nice gesture but can contribute to drug resistance - It is possible that you are inviting another and/or bigger problem | - Having similar symptoms does not mean you both have the same condition - It takes a lot more than just condition to provide an effective prescription. - Patients should understand that appropriate decisions on which medicines to use should be informed by proper diagnostics | - Consulting a qualified medical personnel or healthcare provider is essential to effectively respond | - ***Challenges to following prescription***  *R4: Yes, there is this problem of sharing drugs in the community. Instead of them coming for their dose of medicine, they come to you to request for yours. This makes one not to complete their dose* (**FGD01Craegivers, HC01**)  -  R7: *Then secondly there is something called sharing of drugs. There are people who don’t want to come to the health centre but since we both have headache, that medicine may at the end be used by a husband and wife. So, find that this person doesn’t finish her dose (*(**FGD01Caregivers, HC01**)) |
| Patients are motivated to go to the health center because of the assurance that the right diagnosis will be made after tests performed. | - Always sending the patients to the laboratory before prescription influences adherence. - Patients prefer going to the health facility in place of clinics because of the ability to perform tests - They should know which testing equipment is available for the relevant tests. - Patients get frustrated when the required tests cannot be performed | - Ensure all patients are sent to the laboratory - Ensure the right tests are performed - Communicating to patients before sending them to the laboratory is important in deciding the tests to perform. - Inform patients about the available tests that can be performed at the heath facility | Continuous health talks about the importance of testing before prescription is important   - Refer patients for tests which cannot be performed at the heath facility - Social support is important for patients who are weak | **Motivators to following adherence to prescription**  *R2: What makes it easy, because if you come here, the health workers name the disease that you are suffering from like malaria or typhoid and the medicine they have prescribed are from whatever results they find, because by the time they prescribe for you the medicine, they would have already tested and you take the medicine with confidence (****FGD2 patients HC 02).***  *R: Actually, we know fever manifests in many conditions, we have to go and ensure that they have done the right tests. To find out, is it a malarial cause? Is it a bacterial cause? If we find that it is a malarial cause, then we treat it according to the cause. If we fid that it is maybe due to a bacteria. The bad thing we do not have CBC machine to show us what kind of infection has it occurred but generally the clinician has to prescribe* ***(IDI 002 Nursing officer HC 02)*** |
| Patients’ experiences with health workers/health care seeking at facilities | - Health workers are humans. A whole range of issues and related destructions inform their interaction with the patient and may determine the experience. - It can mediate patients’ understanding of instruction and affects adherence to the prescription s the instructions | - Talk to the health worker supervisor about your experience with the worker if you felt it was inappropriate. You could save both the worker, yourself and other patients - Ask to see another staff if possible, to get more clarity | - The Health facility management committee - Immediate supervisors | ***Barriers to following adherence to prescription***  *R5: I always come to Health Centre2. I then explain the issues I came for to the Clinicians. They then take the history on the book that I will have provided them. Thereafter, they do undertake a blood test and they submit it to the laboratory. They then inform me to proceed to the dispensary after the results are presented to me. Sometimes, when you get to the dispensary, you are told that the drugs or medicines are out of stock thus the need to purchase them from the drug stores or clinics around* **(FGD1 patients HC03)**  ***Barriers to adhering to prescription***  *R7: Sometimes you come to the HC early and find the Health Workers around, after receiving the drugs, say 5 tablets. The 5 tablets will be provided to you, but you will not be informed or explained how to take the medicines. This makes it hard to take medicines* **(FGD4, House hold, HC 02)** |

**Key**

| **HC 01** | Nagongera health centre |
| --- | --- |
| **HC 02** | Aduku health centre |
| **HC 03** | Kihihi health centre |
